# The ‘discontinuity hypothesis’ of depression in later life—clinical and research implications

**DOI:** 10.1093/ageing/afad239

**Published:** 2023-12-28

**Authors:** Richard C Oude Voshaar

**Affiliations:** University of Groningen, University Medical Center Groningen, Department of Psychiatry, Groningen, The Netherland

**Keywords:** depressive disorder, depression, nosology, classification, aged, aged, 80 years and over, older people

## Abstract

The term *depression* is overused as an umbrella term for a variety of conditions, including depressed mood and various psychiatric disorders. According to psychiatric diagnostic criteria, depressive disorders impact nearly all aspects of human life and are a leading cause of disability worldwide. The widespread assumption that different types of depression lie on a continuum of severity has stimulated important research on subthreshold depression in later life. This view assumes that depressed mood is a precursor of a depressive disorder. The present narrative review argues why in later life depressed mood might either (i) lie on a continuum with depressive disorders among people vulnerable for a depressive disorder or (ii) be an ageing-related epiphenomenon of underlying physical illnesses in people who are resilient to depressive disorders (‘discontinuity hypothesis’). Three arguments are discussed. First, the course of depressed mood and depressive disorders differs across the life span. Second, screening instruments for depression have low predictive value for depressive disorders in later life. Third, a dose–response relationship has not been consistently found across different types of depression and detrimental health outcomes. Using the umbrella term *depression* may partly explain why pharmacological treatment is less effective with increasing age, and negative health-related outcomes might be overestimated. The discontinuity hypothesis may prevent pharmacological overtreatment of milder subtypes of depression and may stimulate comprehensive multidisciplinary assessment as well as the development of separate treatment algorithms for depressed mood and depressive disorders.

## Key Points

- Distinguishing between depressed mood and depressive disorders according to diagnostic criteria is crucial for preventing overtreatment and iatrogenic harm.
- Depression screening instruments or fully structured diagnostic interviews might overestimate the prevalence of depressive disorders in later life and their association with ill health.
- In later life, clinical judgement of somatic-affective symptoms is necessary for accurate diagnosis procedures and requires the use of semi-structured diagnostic interviews.
- Procedures for diagnosing depression and depressed mood might benefit from a comprehensive geriatric approach.
- *Watchful waiting*, psychosocial intervention, physical exercises and psychotherapy might be preferable to antidepressant medication for depressed mood in later life.
- Randomised controlled trials on later-life depression should be stratified according to diagnostic subtypes and vulnerability markers for depressive disorders.

## Introduction

The term *depression* is overused in the scientific literature as an umbrella term for either a single symptom of depressed mood, a multidimensional construct assessed with rating scales [1] or a syndrome identified according to diagnostic criteria [2]. These operationalisations are often used interchangeably, which hampers interpretation of clinical-epidemiological studies. Box 1 defines the different types of depression as they are used in this manuscript. Because definitions of depression are not mutually exclusive, we sometimes use the umbrella term *depression* to avoid incorrectly citing articles that include more than one definition.

Using different operational definitions of depression interchangeably implicitly assumes that depression is a dimensional construct ranging from having mild symptoms to having a full-blown depressive disorder. This widespread assumption has stimulated important research on the clinical relevance of milder subtypes of depression in later life [3]. A systematic review on the milder subtypes of depression reported a high prevalence in later life and an association with adverse health outcomes, prompting the authors to recommend more research on the neurobiology and treatment of these subtypes [3]. An important limitation was the heterogeneity in the definitions of the milder subtypes of depression. This precluded the possibility of a meta-analysis and also seemed to question the robustness of the findings because some of the definitions of depression may have resulted in spurious associations.

**Box 1:** Operationalisations of late-life depression

**Name**	**Operationalisation**
Depression	Umbrella term encompassing all definitions and operationalisations of depression, ranging from a single symptom of low mood to a psychiatric disorder according to diagnostic criteria
Depressed mood	A single symptom describing the experience of a low mood or feeling sad
Depressive symptoms^a^	Number and/or severity of several symptoms and/or signs typically related to a depressive disorder according to psychiatric classifications and usually measured dimensionally with self-report and/or observer-rated questionnaires
Clinically relevant depressive symptoms (CRDS)^a^	A sum score above a validated cut-off (against major depressive disorder as gold standard) on a validated screening questionnaire for depressive symptoms
Adjustment disorder with depressed mood	Depressed mood that is (i) related to a stressor, (ii) out of proportion to expected reactions to the stressor and (iii) causes significant distress for the person or impaired functioning
Minor depression (or minor depressive disorder)	DSM-IV-TR term: a psychiatric disorder according to DSM-IV-TR research criteria, i.e. meeting the core symptoms of depressive disorder (low mood and/or anhedonia) and a total of 2–4 criteria, but with subjective distress or functional impairment *Note:* In epidemiological studies, it is also defined as a score above a cut-off (in fact CRDS), sometimes augmented with (explicitly) not meeting criteria for a major depressive disorder
Depressive disorder with insufficient symptoms	DSM-5 term: A psychiatric disorder according to DSM-5 criteria, i.e. falling one-to-three criteria short for a depressive disorder, but still meeting the criterion for subjective distress or functional impairment
Subthreshold depression	A clinical condition falling short on one or more criteria for meeting a formal diagnosis for depressive disorder according to either DSM or ICD criteria
Depressive disorder (or major depressive disorder)	A psychiatric disorder according to either DSM-5 or ICD-11 criteria; often called *major depressive disorder*
Dysthymia	A mild depressive disorder with chronicity >2 years according to DSM-IV-TR criteria
Persistent depressive disorder	A depressive disorder with a protracted course of at least 2 years according to DSM-5 criteria

^a^ *Note*: Depressed mood (or anhedonia) is a core symptom of all syndrome operationalisations. Nonetheless, *depressive symptoms* (dimensional) or *clinically relevant depressive symptoms* (exceeding a cut-off value) are based on either self-report or observer-rated scales and do not necessarily signify depressed mood or anhedonia.

Considering a continuity between depressed mood and depressive disorder, however, matches the dimensional approach to psychopathology as described in the Hierarchical Taxonomy of Psychopathology (HiTOP) initiative [4]. This approach identifies psychopathology constructs based on patterns of co-variation among symptoms [5]. The HiTOP approach might help in the identification of universal risk factors by linking them to transdiagnostic spectra [5]. Arguments for a dimensional approach to depression often rely on studies that have identified the same determinants and health consequences in milder subtypes of depression as in depressive disorder [6]. Nonetheless, a dimensional approach does not (i) offer a solution to the multifactorial nature of psychiatric disorders, (ii) identify the relative contribution of specific risk factors in individual patients or (iii) acknowledge the periodicity of psychiatric disorders [7]. Regarding depression in later life, potential changes in the relative contribution of different risk factors that accompany ageing are not addressed [7]. Neither is the differential impact of risk factors along the degree of severity of the depression, nor the potential discontinuity between depressed mood and depressive disorders.

The present narrative review critically evaluates arguments as to why a depressed mood or depressive symptoms might not necessarily lie on a continuum with depressive disorders in later life. It also addresses how this discontinuity hypothesis might inform clinical practice and future research in geriatric psychiatry.

## Methodology

This narrative review is based on a literature search of systematic reviews and meta-analyses on depression in later life. Keywords related to *depression* and *old age* were combined with the terms *systematic review* and *meta-analysis* to search for articles in *PubMed* up until 1 July 2023. This yielded 2,291 hits. Reviews relevant to the objectives of the present paper were extracted and reviewed in detail. In addition, individual papers that were cited in the reviews were extracted. Publications on bipolar depressive disorder were considered outside the scope of this review.

## Arguments supporting the discontinuity hypothesis

In this section, we discuss the empirical findings that support the discontinuity hypothesis, and we indicate that depressed mood and depressive disorder might become separate pathways with ageing.

### Epidemiological findings

#### Differential course of depressive disorders during ageing

It has long been thought that depressive disorders have their highest prevalence in older age groups, but compelling evidence from epidemiological studies now demonstrates the opposite [8]. Among 146,315 people aged 18 to 80, advanced non-linear models showed that the prevalence of depressive disorders reached a plateau between the ages of 30 and 50 and decreased thereafter [9]. By contrast, depressive symptomatology was more stable across the lifespan and even increased after the age of 65 [9]. This study is consistent with incidence studies, which show that lower incidence rates with increasing age disappear when depression is not defined as a depressive disorder, such as dysthymia or minor depression [10–12]. The different trajectories of milder subtypes of depression and depressive disorder according to age suggest that a depressed mood has a different meaning and a different origin in later life than in younger age groups, and it less often progresses into a depressive disorder. Otherwise, the absolute and relative contribution of a depressed mood as a risk factor for depressive disorder differs according to age.

#### Low predictive value of screening instruments in older ages

Considering that late-life depressive disorders are common and on a continuum with depressed mood, one would expect that cut-off values on depression rating scales would have high positive predictive value (PPV) for depressive disorder. Self-report screening questionnaires, however, overestimate the prevalence of depressive disorder [13]. Cut-off scores on the 15-item Geriatric Depression Scale (GDS-15) or on the Hospital Anxiety Depression Scale (HADS) were at best in moderate agreement with the diagnosis of a depressive disorder based on a semi-structured diagnostic interview (Kappa values ranged from 0.22 to 0.31) [14]. Furthermore, the PPV from self-report depression screening questionnaires is rather low, with reported values as low as 13 and 23% for depressive disorder (but negative predictive values of 96 and 99%) [15, 16]. These values show that these scales are optimised to exclude the possibility of a depressive disorder, whereas in epidemiological studies these scales are generally used as proxies for a depressive disorder. Misclassification biases might be particularly problematic in geriatric psychiatry because false positives are associated with increasing age, physical illness, disability and cognitive decline [16–18]. A low PPV may indicate confounding resulting from underlying somatic illnesses and inflate prospective associations between depression as assessed with self-report scales and negative health outcomes.

#### Absence of a dose–response relationship in old age

A dimensional approach to depression in later life assumes a dose–response relationship with negative health-related outcomes. Consider mortality, the ultimate adverse outcome. Meta-analysis of population-based studies did not found that the increased mortality risk differed significantly between people suffering from a depressive disorder and those with clinically relevant depressive symptoms or minor depression [19]. Another meta-analysis showed that the relative risk of mortality associated with depression did not differ between 166 studies that used self-report scales (RR =1.64, 95% CI = 1.49–1.80) and 72 studies that used a diagnostic interview (RR = 1.64, 95% CI = 1.56–1.73) [20]. Symptoms of physical illnesses may be picked up by self-report depressive symptom scales and confound associations of milder types of depression with mortality resulting in similar strengths as found for depressive disorders.

Among clinical samples of depressed patients, even inverse associations have been reported. For instance, in the Netherlands Study of Depression in Older people, mortality rates that were adjusted for health-related behaviours and morbidity were significantly higher among those suffering from minor depression compared to those suffering from a depressive disorder [21]. One explanation is that minor depression in later life also reflects symptoms due to ageing-related processes, such as physical and cognitive impairments that are associated with mortality [21]. Among 4,243 patients aged ≥65 and diagnosed with depressive disorder, a higher mortality risk was associated with proxies of biological ageing [22]. Conversely, in this latter study core features of depression were associated with lower mortality risk [22].

Collectively, these findings seem to point to biased estimates due to confounding by underlying physical illnesses.

### Clinical findings

#### Depression as an epiphenomenon of physical illnesses

Non-specific symptoms related to physical ageing or physical illness, such as fatigue or concentration problems, may easily be classified as symptoms of a depressive disorder. In the AgeMooDe study of 1,197 participants aged 75+ years, exceeding the cut-off for either the GDS-15 or the HADS was associated with greater disability than that of clinical diagnosis of depressive disorder [14]. This may indicate an overestimation of depression with the GDS or the HADS due to chronic disease-related disability.

Because ageing-related physical changes are associated with low mood and anhedonia [23] as well as with adverse health effects [24], self-reported depressive symptoms may inflate negative health outcomes in mild cases of late-life depression. To illustrate the potential impact of this issue, Table 1 summarises the most recent meta-analyses on adverse health outcomes of late-life depression. Only a minority of studies (median = 25%) included in these meta-analyses have assessed depression according to psychiatric diagnostic criteria [25–31].

**Table 1:** Depression as a predictor for adverse health outcomes

**Study (authors, year)**	**Adverse health outcome**	**Proportion of studies based on MDD**	**Heterogeneity due to assessment of depression**
Luppino et al. (2010)	Obesity	6/10 (60.0%)	Numerical strongest association with depressive symptoms, but not significantly different from depressive diagnosis
Meng et al. (2012)	Hypertension	3/9 (33.3%)	Explored, but invalid due to limited number of studies
Yu et al. (2015)	Diabetes	5/33 (15.2%)	Not examined
Miloyan & Fried (2017)	Mortality	50/293 (17.1%)	Not explicitly examined. Only 4/293 studies met prior quality indicators; pooling these studies revealed no significant association
Wang et al. (2020)	Cancer	5/20 (25.0%)	Subgroup analyses showed only significant risk among those with a clinical diagnosis, but difference between the two types of assessment was not significant (*P* = 0.27)
Cao et al. (2022)	Coronary heart disease	5/26 (19.2%)	Not examined
Stafford et al. (2022)	Dementia	15/33 (45.5)	Not examined

For instance, after a myocardial infarction, patients’ depressive symptom severity is inversely associated with the left ventricular ejection fraction [32], and low scores on depression scales specifically indicated an underlying somatic illness [33]. Furthermore, frailty and depression may easily be confused due to overlapping diagnostic criteria [34], but the overlap with depressive disorder is still less compared to self-report depressive symptoms [35]. Even though more than 280 rating scales have been developed to assess depression [36], none of them has satisfactorily solved this problem. Paradoxically, depression rating scales designed for older people (e.g. the GDS-15) exclude or minimise the importance of somatic-affective symptoms, although these symptoms are intrinsically related to depressive disorders, and depressive disorders have a more somatic presentation in later life [37]. Therefore, appropriate clinical evaluations of somatic-affective symptoms are needed in clinical practice [38].

#### Depression as an epiphenomenon of neurodegenerative disorders

Whether depression is a causal factor in dementia or a prodrome (and thus an epiphenomenon) of neurodegenerative processes is unclear. The two possibilities are, however, not mutually exclusive [39]. Arguments for a causative relationship are as follows: (1) there is a dose–response relationship between depression severity and the onset of dementia, and (2) early-onset depression is associated with dementia [29]. A Danish register-based cohort study that included almost 600,000 men supported the second argument by showing that midlife depression doubled the risk of dementia in later life [40]. Arguments supporting the hypothesis that depression is a prodrome of dementia are as follows: (i) there are stronger associations with late-onset versus early-onset late-life depression; and (ii) stronger associations have been reported in studies with shorter follow-up periods [29]. Longitudinal studies with repeated assessments of depressive symptoms have also supported the prodromal hypothesis. In the Rotterdam scan study, increasing levels of self-reported depressive symptoms across 10 years predicted the onset of dementia, whereas consistently elevated self-reported depressive symptoms did not [41]. In the Whitehall-II study, which included over 10,000 participants, depressive symptoms across 28 years were analysed, and results showed that differences in self-reported depressive symptoms between those with and without dementia became apparent 11 years before the onset of dementia and were nine times larger during the year of the diagnosis [42]. These different pathways would appear to warrant different treatment approaches.

## Mechanisms underlying the discontinuity hypothesis

A shift in etiological mechanisms for depression occurring with ageing might explain why the prevalence of milder subtypes of depression increase with ageing, but depressive disorders decrease with ageing.

Meta-analysis of genome-wide association studies has concluded that ‘all humans carry lesser or greater numbers of genetic risk factors for depressive disorder, which implies that a continuous measure of risk underlies the clinical phenotype’ [43]. Genetic risk, however, might differ among the different subtypes of depression. In three large twin samples, total heritability differed little between depressive disorders (0.31) and depressive symptoms (0.30), while the proportion of genetic risk that was unique for life-time depressive disorders was 65% but for depressive symptoms 0% [44]. In other words, a depressed mood or depressive symptoms are not adequate proxies for the genetic vulnerability to a depressive disorder [44]. Furthermore, the inverse association between age of onset of a depressive disorder and genetic risk factors for a depressive disorder [43, 45] implies that other risk factors become more important in later life or that new risk factors emerge with a later onset.

Vulnerability for the different types of depression, however, includes more than genetics [46]. It is often assumed that resilience increases with age because of lower prevalence rates of mental illnesses in old age [47]. Meta-regression analyses have shown better emotion-regulation strategies with increasing age, but no study has included participants older than 65 [48]. More recent studies comparing younger and older (65+) adults have found that older adults become especially adept at suppressing negative affect [49, 50]. An alternative hypothesis for the low rates of psychopathology in later life is the *on-time*, *off-time* hypothesis for explaining psychosocial stressors. Although stressors such as loneliness, bereavement and physical disabilities are by far more prevalent in later life, these same stressors might trigger higher stress levels in younger age groups [7]. Nonetheless, older people may still respond to these stressors with a depressed mood, and this would explain the highest prevalence of the milder subtypes of depression in the oldest age groups.

**Figure 1:**
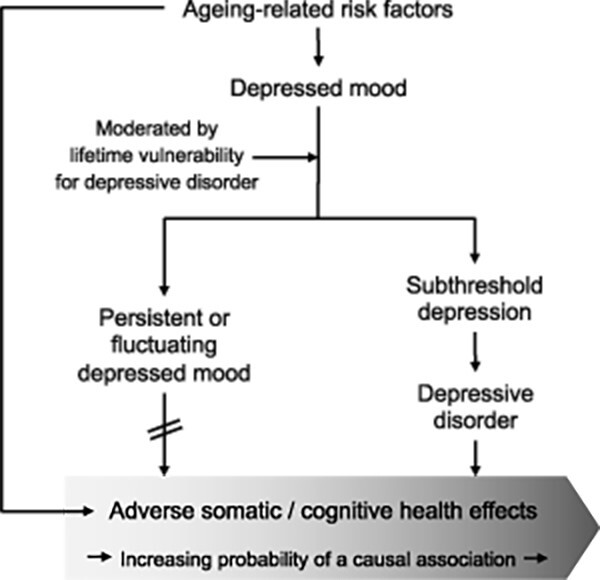
Conceptualisation of the ‘discontinuity hypothesis’. *Note*: Age-related risk factors can contribute to a depressed mood. Among older individuals resilient to depressive disorder, their mood issues may not progress to a depressive disorder, whereas those vulnerable to depressive disorder face an increased risk of developing one. Although the figure suggests two mutually exclusive pathways, vulnerability and risk factors are dimensions that may partially overlap. Patients vulnerable to depressive disorder may also be at an increased risk of adverse somatic and cognitive health outcomes. Improving mental health throughout one’s life can enhance healthy ageing. For those experiencing a depressed mood due to age-related risk factors, the impact of such mood issues on adverse health outcomes might be a spurious association, explained by the direct effect of these age-related risk factors. Therefore, both risk factors and mood problems should be addressed in their case.

During the past decades, vascular ageing [51], cognitive ageing (neurodegeneration) [52], physical ageing (frailty) [53], dopamine dysfunction [54] and low-grade inflammation (‘inflammageing’) [55] have received attention as risk factors for all subtypes of depression, and they are likely to become proportionally more relevant with ageing [7]. Whereas ageing mechanisms affect all older people to some extent, only a minority of them become clinically depressed. One might hypothesise that the ageing-related mechanisms result in a depressed mood that may or may not progress into a depressive disorder in an individual depending on their vulnerability for depressive disorder (Figure 1).

Even though the age-related mechanisms may often result in a depressed mood instead of a depressive disorder, it is important to acknowledge that in clinical practice, the mechanisms might be highly relevant. This is shown by the fact that although the median age of onset of a depressive disorder is 30 years (IQR 21–44) [56], consecutive sampling of depressed older patients in specialised mental health care shows that about half of them suffer from a late-onset depressive disorder [57–59].

To study these ageing-related mechanisms in-depth, studies should include both older people with depressed mood and those with a depressive disorder to learn how the mechanisms interact with vulnerability for a depressive disorder.

## Depression as a geriatric syndrome

In contrast to medical syndromes, geriatric syndromes are not characterised by symptoms that collectively indicate a disease, but refer instead to multiple factors that are (i) prevalent in older adults and (ii) associated with adverse health outcomes [60]. Geriatric syndromes often refer to a single symptom, which does not represent one underlying pathophysiology, e.g. falls or urinary incontinence [61]. Late-life depression meets the definition of a geriatric syndrome; it is a common and multifactorial condition, which places an individual at increased risk of adverse health outcomes. Nonetheless, geriatric medicine may benefit from a better distinction between depression as a geriatric syndrome and depression as a psychiatric disorder according to diagnostic criteria.

### Clinical relevance

When depressed mood originates from age-related pathophysiological processes or disability, one may assume a more chronic course and lower responsiveness to treatment.

#### Limited efficacy of antidepressants for conditions other than a depressive disorder

Antidepressants are not effective for milder forms of depression [62]. Treating minor mood changes in frail older adults with antidepressants may even be harmful as even minor side effects may trigger adverse health effects [24]. Furthermore, having residual symptoms following treatment for depression may in some patients be an epiphenomenon of an underlying somatic illness [38]. Because antidepressants do not target age-related mechanisms, this might be why the effectiveness of antidepressants decreases with increasing age [63] and why the natural course of depression is less favourable and spontaneous remission is less likely among older adults [64, 65].

#### Overtreatment by focussing on depressed mood

Over-prescription of antidepressants is a timely topic. A medical records-linkage study with multidisciplinary expert review of antidepressant prescriptions found 24% of 3,199 new prescriptions for older people (65+) as potentially inappropriate. Over-prescription largely involved non-specific psychiatric symptoms and subthreshold diagnoses [66]. Another study, which concluded that over-prescribing of antidepressants was not a problem, found that 18% of 7,051 patients starting antidepressant treatment had low or mild depressive symptoms, ranging from 11% in patients <30 to 26% in patients ≥65 [67]. Whether the data are acceptable or alarming, potentially inappropriate prescribing consistently increases with age [67, 68]. To improve milder subtypes of depression in later life, several evidence-based options are available. Meta-analyses of randomised controlled trials have shown that both home-based interventions for reducing loneliness in later life, reminiscence therapy and physical exercise ameliorated depressive symptoms [69–71].

In the clinical geriatric assessment (CGA)-Swed study, 61% of frail older adults with unplanned hospital admissions suffered from clinically relevant depressive symptoms based on cut-off scores of an observer-rated depression scale [72]. Those randomly assigned for a CGA were more often prescribed antidepressants than those assigned to usual care. Although this was interpreted as positive, i.e. a reduction of undertreatment due to better case management [72], one might question whether pharmacological treatment for clinically relevant depressive symptoms is appropriate. In the Longitudinal Ageing Study Amsterdam, for example, only 15% of the patients who scored above a cut-off score on the Center for Epidemiologic Studies Depression Scale developed a depressive disorder during a 17-year follow-up, whereas 40% of them remained stable, and 45% recovered [73]. These figures argue for *watchful waiting* instead of immediately starting antidepressant treatment. Furthermore, the risk of falling due to antidepressant medication exponentially increases when patients are frail [74]. Because the item *being unable to do things that make one feel valued* was the only independent correlate of clinically relevant depressive symptoms in the CGA-Swed study, psychological interventions might have been a better treatment option [46, 53]. Whereas studies of older adults are still limited, meta-analyses thus far point to similar or even higher efficacy for psychotherapy for depression with increasing age [75]. Outside the hospital, in two-thirds of the patients presenting with clinically relevant depressive symptoms, antidepressants were initiated during the first consultation [76, 77]. Most older adults, however, prefer psychological support [78].

#### Screening and prevention

Screening for depression is not always advised. Although the US Preventive Services Task Force advocates depression screening with the GDS for older adults [79], the Canadian Task Force on Preventive Health Care advises against screening for depression in general. This latter view is based on the lack of positive findings in randomised controlled trials, particularly in the case of collaborative care. Collaborative care may not be beneficial for patients who screen positive [80]. First, many patients are already being treated. Second, accuracy in identifying previously unrecognised patients is low. Third, treatment effects for less severe depression are relatively small [80]. These arguments may be particularly valid for depressed older persons, as the proportion of depressed people not meeting the criteria for a depressive disorder increases with increasing age [9].

The incidence of a depressive disorder among older adults with milder subtypes of depression is estimated to be at least 10% per year [3]. Therefore, prevention targeting people with depressed mood or elevated depressive symptoms may be effective [81]. In fact, a meta-analysis has shown that indicated prevention of late-life depression is effective [82], with a number needed to treat of 15 for psychotherapy [83].

#### Accurate measurement of depressive symptoms

Meta-analyses comparing treatment effects have revealed smaller effect-sizes when outcomes are based on self-reported versus clinician-rated scales [84]. This might suggest that clinicians can better distinguish between low mood as a symptom of a depressive disorder and low mood as an epiphenomenon of chronic somatic conditions. Clinician-rated scales are indeed considered the gold standard. Application in epidemiological studies, however, is generally too expensive since clinical experience is mandatory.

## Methodological considerations

This narrative review is not intended to be a systematic literature review. Instead, it is a selective presentation of the literature to substantiate that the prevalence and negative health outcomes of late-life depression may be overestimated as a result of considering depressed mood to be at the lower end of a continuum with depressive disorders at the other end. A better understanding of clinical experience is important because the epidemiological literature generally emphasises undertreatment of late-life depression but hardly addresses iatrogenic damage due to overtreatment of depressed mood.

Most findings that argue for a discontinuity between depressed mood and a depressive disorder in later life provide only indirect evidence. Even though a depressed mood might be considered a geriatric syndrome on its own in older persons who are resilient to a depressive disorder, it may still lie on a continuum with depressive disorders in a subgroup of patients (see Figure 1). This review provides some evidence that clinical judgement might help to distinguish between the two types of depressed mood, but further research is needed to determine the extent to which this is feasible in clinical practice. In addition, the distinction between depressed mood and *normal* mood is arbitrary and probably dictated more by socio-cultural norms than by medical norms.

Furthermore, this review has focused primarily on the impact of physical functioning and biological ageing as driving factors underlying depressed mood. The model may also apply to people with neuroticism. Depressive disorders share less than half of their genetic variance with neuroticism [44], and people with only a unique genetic liability for neuroticism but not for a depressive disorder may suffer from low mood without being at increased risk for a depressive disorder.

## Implications for clinical care and future research

The discontinuity hypothesis of depression in later life has both clinical and research implications.

First, the high prevalence of a depressed mood in later life requires treatment to improve sufferers’ well-being. Treatment algorithms for depressed mood are, however, not the same as for depressive disorders. It is unlikely that a depressed mood will progress into a depressive disorder in older people who are not vulnerable to developing a depressive disorder. This is particularly true for older people for whom the pathophysiology is driven by age-related risk factors (see Figure 1). *Watchful waiting* might be indicated and antidepressants contra-indicated to prevent iatrogenic damage. Furthermore, positive psychology might be preferred for targeting depressed mood [53], like life review, acceptance and commitment therapy, and mindfulness-based cognitive therapy.

Second, stratification of clinical trials is needed to empirically investigate many of the assumptions presented in this review. Clinical trials targeted at a broad range of subtypes of late-life depression require stratified randomisation according to diagnostic subtype. Clinical trials for people with milder forms of depression, who need support to improve their mood, should stratify randomisation according to long-standing vulnerability markers for depressive disorders.

Third, people with a depressed mood might be more reactive to external circumstances than those meeting the diagnostic criteria for a depressive disorder [2]. Assessing day-to-day variability in mood using experience sampling methods (ESM) may be particularly relevant for those who do not meet the criteria for a depressive disorder. ESM generally uses mobile technology to study what people do, feel and think during their daily lives by randomly asking them to self-report throughout the day. These data allow for close monitoring with high ecological validity to identify early warning signals of mood deterioration and to identify person-specific determinants of mood changes [85–87]. An ageist attitude often leads to exclusion of older persons from ESM studies, while this may only apply to older persons with (mild) cognitive impairment [88–90].

Fourth, the complex etiological heterogeneity of late-life depression warrants a geriatric approach. The traditional biological, psychological and social factors indicate patients’ vulnerability for a depressive disorder and their age-related risk factors for depressed mood. Together, they can be used to estimate the patient’s risk for progression to a depressive disorder (see Figure 1). Semi-structured diagnostic interviews for diagnosing depressive disorder and clinician-rated severity scales are mandatory in both clinical practice and high-quality research in late-life depression [91]. Semi-structured interviews are also able to take the periodicity of depressive disorder into account by assessing past and current episodes. Self-report depression scales could be added to allow differentiation between the detrimental health consequences of a depressive disorder and depressive symptoms as an epiphenomenon.

Fifth, differences between older and younger age groups should be empirically evaluated. Future studies should examine whether genetic risk factors for depression are moderated by age or differentially interact with environmental factors across the life span. Randomised controlled trials on treatment and prevention of depressive disorders should include participants with a broad age-span. Age-stratified randomisation with sufficient statistical power in all age groups is needed to examine differential effects between younger and older people. The same holds true for epidemiological and clinical cohort studies on depression. Epidemiological studies should examine the interaction between age-related risk factors for depressed mood and vulnerability for a depressive disorder. Because the prevalence of a determinant and the strength of its association with a depressive disorder may differ across the lifespan [92], studies are needed to refine age-specific prevention and treatment algorithms.

## Conclusions

Late-life depression is an important topic that needs to be addressed using a coordinated and tailored approach to health and social care. This requires a proactive approach for monitoring and reviewing the mood of older adults, acknowledging that depressed mood and depressive disorders do not necessarily lie on the same continuum of severity. To prevent overtreatment, proper diagnostics conducted by well-trained physicians are needed instead of only self-report instruments and structured interviews. Considering late-life depression as a geriatric syndrome may bring the spotlight onto this problem and send a clear signal to geriatric experts that depression is highly heterogeneous and deserves a more personalised approach.
